# Simultaneous Analysis of Mycotoxins, Potentially Toxic Elements, and Pesticides in Rice: A Health Risk Assessment Study

**DOI:** 10.3390/toxins15020102

**Published:** 2023-01-20

**Authors:** Mohammad Hashem Yousefi, Esmaeel Abbasi, Milad Hadidi, Seyedenayat Hashemi, Amir Hossein Ghadimi, Saeed Yousefinejad, Hossein Arfaeinia, Abbas Yousefinejad, Przemysław Łukasz Kowalczewski, Agnieszka Tomkowiak, Saeid Hosseinzadeh, Amin Mousavi Khaneghah

**Affiliations:** 1 Department of Food Hygiene and Public Health, School of Veterinary Medicine, Shiraz University, Shiraz 71946-84471, Iran; 2 Department of Organic Chemistry, Faculty of Chemical Sciences and Technologies, University of Castilla-La Mancha, 13071 Ciudad Real, Spain; 3 Department of Environmental Health Engineering, Faculty of Health and Nutrition, Bushehr University of Medical Sciences, Bushehr 75146-33341, Iran; 4 Department of Food Science and Technology, Yasooj Branch, Islamic Azad University, Yasooj 75914-93686, Iran; 5 Research Center for Health Sciences, Institute of Health, School of Health, Shiraz University of Medical Sciences, Shiraz 71348-14336, Iran; 6 Energy Research Center, Bushehr University of Medical Sciences, Bushehr 75146-33341, Iran; 7 Department of Nutrition, Sarvestan Branch, Islamic Azad University, Sarvestan 73541-94579, Iran; 8 Department of Food Technology of Plant Origin, Poznan University of Life Sciences, 31 Wojska Polskiego St., 60-624 Poznan, Poland; 9 Department of Genetics and Plant Breeding, Poznan University of Life Sciences, 11 Dojazd St., 60-632 Poznan, Poland; 10 Department of Fruit and Vegetable Product Technology, Prof. Wacław Dąbrowski Institute of Agricultural and Food Biotechnology—State Research Institute, 36 Rakowiecka St., 02-532 Warsaw, Poland; 11 Department of Technology of Chemistry, Azerbaijan State Oil and Industry University, 16/21 Azadliq Ave, Baku AZ1010, Azerbaijan

**Keywords:** mycotoxin, PTE, rice, risk assessment, pesticide

## Abstract

Rice is a widely consumed food worldwide; however, it can be a source of pollutants, such as potentially toxic elements (PTEs), mycotoxins, and pesticides. Sixty rice samples imported from Pakistan (PAK), India (IND), and Thailand (THAI), as well as domestic Iranian (IRN) rice, were collected from Bushehr, Iran, and investigated for the contamination of PTEs, including arsenic (As), lead (Pb), cadmium (Cd), and nickel (Ni); pesticides, including chlorpyrifos, trichlorfon, diazinon, fenitrothion, and chlorothalonil; mycotoxins, such as aflatoxin B1 (AFB1), zearalenone (ZEN), ochratoxin A (OTA), and deoxynivalenol (DON); and molds. Estimated daily intake (EDI) and hazard quotient (HQ) of pollutants and hazard index (HI) and incremental lifetime cancer risk (ILCR) of rice types for the Iranian adult population were calculated. The content of PTEs in Iranian rice was not higher than Iran’s national standard limits. In contrast, other types of rice (imported) had at least one PTE above the permissible level. OTA content was below the detection limit, and all other mycotoxins were within the allowable range in all rice types. Thai rice was the only group without pesticides. The HI order of rice types was as follows: HI_PAK_ = 2.1 > HI_IND_ = 1.86 > HI_IRN_ = 1.01 > HI_THAI_ = 0.98. As was the biggest contributor to the HI of Iranian and Thai rice, and diazinon in the HI of Pakistani and Indian rice. The calculation of ILCR confirmed that the concentrations of Ni and Pb in Pakistani and Ni and As in Indian, Thai, and Iranian rice were not acceptable in terms of lifetime carcinogenic health risks.

## 1. Introduction

Rice (*Oryza sativa*) is a popular staple food and is frequently consumed by the world’s population [1,2]. This commodity is rich in essential nutrients, such as proteins, carbohydrates, and vitamins, and provides two-thirds of caloric requirements [3,4]. Since domestic rice production is insufficient in Iran, part of the national need is imported from the biggest rice producers, such as Pakistan and India. According to Faostat, the per capita rice consumption in Iran, Thailand, Pakistan, and India is 42.6, 178, 17.1, and 103 kg, respectively, in 2021 [5].

About 20 mycotoxins, including aflatoxins, ochratoxin, zearalenone, and deoxynivalenol, are frequently present in foodstuffs and animal feed and are produced by fungi, such as *Aspergillus*, *Penicillium*, and *Fusarium* [6,7,8,9,10,11,12]. Mycotoxins can cause serious health consequences, such as liver cancer, nephrotoxicity, and renal cancer, in children with premature sexual development and disorders in the gastrointestinal tract, immune, reproductive, endocrine, and nervous systems [13,14,15,16], respectively.

Other types of contaminants in rice include pesticides, such as insecticides, herbicides, acaricides, and fungicides, that can enter the edible parts of the plant and then the human food chain [17,18]. Rice cultivation is the second consumer of pesticides after cotton [19]. Human oral intake of pesticides can lead to toxicity and chronic consequences, such as genetic mutation and dysfunction and blood, reproductive, and endocrine disorders [20,21,22,23,24,25].

Anthropogenic activities, such as mining, overuse of chemicals, industrial activities, and the use of fossil fuels, lead to the presence of or increase in metalloids and potentially toxic elements (PTEs) in the soil. Then, these pollutants, such as arsenic (As), cadmium (Cd), and lead (Pb), are absorbed and accumulated by rice plants and eventually enter the human food cycle [26,27,28]. As a toxicologically relevant element, Pb has been brought into the environment by man in extreme amounts, despite its low geochemical mobility, and has been distributed worldwide [29]. Cd, which enters the human, can remain in the body for years and, in amounts exceeding the permitted limits, can lead to kidney damage. Lead is a highly toxic metal related to nervous system disorders, weakness in the knees and fingers, and a mild increase in blood pressure. As is associated with skin and lung cancer, peripheral neuropathy, and anemia. Ni also can face humans with carcinogenicity, teratogenicity, and various toxicities, such as genotoxicity, hematotoxicity, and immunotoxicity [30].

The presence of different contaminants in a single matrix (especially in food) requires serious attention due to the interactions they can have with each other. Tsatsakis et al. [31] suggested that different pollutants could have synergistic, combined, or competitive effects that could generate a new risk or hazard. At the same time, they could not indicate that potential risk or hazard alone.

Since rice is an appropriate matrix for a set of chemical co-contaminants (PTEs, pesticides, and mycotoxins), it was necessary to pay attention to cross-contamination risks by conducting this study. This study aimed to determine the amount of co-contaminants in Iranian and imported rice and subsequently to assess the health risks (carcinogenic and non-carcinogenic) caused by its consumption among Iranian adults.

The most important innovative aspect of this study is the comprehensive investigation of all the potential contaminants of rice, including microbial (mold and yeast), heavy metals, pesticides, and mycotoxins, and the risk assessment of its consumption in terms of both non-carcinogenic and carcinogenic effects. According to our information, this study is unique in Iran.

## 2. Results and Discussion

### 2.1. Ash, Moisture, and Mold Contamination

All our groups were in the acceptable range of moisture content suggested by ISIRI (<13.5%) (Table 1). However, India and Pakistan possessed the highest and the lowest moisture content, respectively. In addition, the highest ash contents belonged to the Pakistani group, which is significantly higher than the remaining groups. The highest and lowest mold count was observed in the Indian and Pakistani groups. There was a significant difference between the groups concerning mold contamination. Only the Indian samples had a non-acceptable mold count regarding ISIRI permissible limit (10^4^ CFU g^−1^). The Pakistani group with the lowest mold count was the only group that possessed the fungicide (chlorothalonil). Aydin et al. [32] reported a range of 1.0 × 10^1^ to 1.5 × 10^4^ CFU g^−1^ of mold count among Turkish rice.

### 2.2. PTEs

As Table 2 describes, the As contamination rate of all imported and domestic rice brands were not exceeding Iran’s national standard limit (0.15 mg kg^−1^). However, the highest and the lowest level of As contamination belonged to Iranian and Pakistani samples, respectively. The As the content of Pakistani brands (0.045 ± 0.019 mg kg^−1^) was significantly lower than the Iranian and Thai brands. Almutairi et al. [33] reported a contamination rate of 0.1 ± 0.024 mg kg^−1^ for Indian rice, which is in line with the present results. Another study from Iran showed a higher As rate with an average of 0.369 ± 0.094 mg kg^−1^ [34]. Additionally, in another study from Saudi Arabia, As contents of 0.202 ± 0.041 and 0.183 ± 0.030 mg kg^−1^ in soaked and rinsed rice samples were observed, respectively [35]. Similar results (As average content of 0.181 mg kg^−1^) were observed in different countries’ originated rice samples in a study by Jallad [36] in Kuwait. Li et al. [37] also observed As contamination rate of 0.155 mg kg^−1^ in rice samples cultivated in northeastern China. Contrary to the As content, the Pb content of the Pakistani samples was significantly higher than the Iranian and Thai samples. The amount of lead in Pakistani and Indian samples exceeded the allowable limit of the National Standard of Iran (0.15 mg kg^−1^), while the Iranian and Thai samples were in the permissible range. Djahed et al. [34] reported a similar result to our study (0.123 ± 0.14 mg kg^−1^). However, some other studies have presented a higher lead concentration, including 11.5 mg kg^−1^ in the north of Iran and 17.13 ± 0.4 mg kg^−1^ in India [38,39]. On the other hand, this rate was much lower in the two mentioned studies of Saudi Arabia, with a lead concentration of 0.023 ± 0.008 mg kg^−1^ in India and also 0.034 and 0.057 mg kg^−1^ in soaked and rinsed samples [33,35]. Moreover, Liu et al. [40] showed a similar lead concentration of an average of 0.031 ± 0.052 mg kg^−1^ in rice samples cultivated in Sri Lanka.

The highest and the lowest concentration of Cd belonged to the Indian and Iranian samples, respectively. With the exception of the Iranian samples, the Cd concentration of all Pakistani, Indian, and Thai samples exceeded the allowable limit of the Iran National Standard (0.06 mg kg^−1^), and it was significantly higher than the Iranian samples. However, in Thai samples, it was more than the permissible limit of ISIRI. The other Iranian study showed a similar Cd content to our Iranian samples (mean of 0.0337 ± 0.039 mg kg^−1^), and Liu et al. [40] also presented a similar result to our remaining samples (0.080 ± 0.130 mg kg^−1^). Almutairi et al. [33] and Al-Saleh and Abduljabbar [35] reported a lower Cd concentration of 0.019 ± 0.005, 0.015, and 0.027 mg kg^−1^ in Indian, soaked, and rinsed samples, respectively. Conversely, Praveena and Omar [41] showed a much higher Cd contamination rate of 0.160 mg kg^−1^ in cooked rice in Malaysia. Additionally, Kormoker et al. [42] reported a range of 0.11 ± 0.04 to 0.24 ± 0.12 mg kg^−1^ of Cd contamination rate in domestic Bangladeshi samples.

Regarding Ni, no allowable limit was set by Iran’s national standard sets, but all the samples were below the permissible limit of FAO/WHO (10 mg kg^−1^). However, the Pakistani and the Iranian samples possessed the highest and lowest Ni concentrations. In addition, a significant difference was observed among all four groups. Kormoker et al. [42] suggested a much higher Ni concentration range of 3.81 ± 3.38 to 13.23 ± 8.31 mg kg^−1^ in Bangladeshi-cultivated rice. On the other hand, Shraim [43] reported an average concentration of 0.064 mg kg^−1^ (0.042–0.086) in only 7% of the rice samples purchased from Almadinah Almunawarah city, Saudi Arabia.

### 2.3. Mycotoxins

#### 2.3.1. Aflatoxin B1

All details of the rate of mycotoxins contamination are presented in Table 3. AFB1 was detectable in Pakistani and Thai samples; however, they did not exceed the ISIRI (5 µg kg^−1^). The AFB1 level of Pakistani samples was significantly higher than the Thai. Almutairi et al. [33] also reported AFB1 contamination below the LOD among their samples, which follows our Iranian and Indian samples. Majeed et al. [44] also observed a rate of 5.84 ± 6.69 µg kg^−1^ in domestic rice samples from Punjab, Pakistan. Another study from Iran reported higher AFB1 contamination rates of 15.13 ± 33.78 and 11.61 ± 29.56 µg kg^−1^ in domestic and imported rice, respectively [45].

#### 2.3.2. Zearalenone

All four groups easily passed the ISIRI permissible limit of the ZEN concentration (200 µg kg^−1^), especially Pakistani samples with no detectable ZEN level. The ZEN content of the Iranian group was the highest, significantly higher than the Indian group. Eslamizad et al. [45] also reported that all their domestic and imported samples possessed ZEN concentrations below the LOD. A contamination rate of 8.46 ± 21.57 µg kg^−1^ was reported by Majeed et al. [44]. Pan [46] presented a range of 2.46 ± 3.88 to 52.93 ± 26.98 µg kg^−1^ in Chinese paddy rice.

#### 2.3.3. Ochratoxin A

All our rice samples had OTA contamination below the LOD; however, Eslamizad et al. [44], Majeed et al. [44], and Pan [46] reported 1.37 ± 5.72, 0.610 ± 2.68, and 0.85 ± 1.2 µg kg^−1^ of OTA in their samples. The permissible limit of ISIRI is set as 5 µg kg^−1^.

#### 2.3.4. Deoxynivalenol

Finally, the last investigated mycotoxin, DON concentration, was in the acceptable range of ISIRI (1000 µg kg^−1^), and the highest and the lowest concentrations belonged to Indian and Iranian samples, respectively. Eslamizad et al. [45] presented a higher DON concentration of 94.64 ± 13.13 and 117.38 ± 24.78 µg kg^−1^ among domestic and imported samples, while Majeed et al. [44] reported a lower concentration of 6.99 ± 22.7 µg kg^−1^.

### 2.4. Pesticides

#### 2.4.1. Chlorpyrifos

All details about pesticide residue are reported in Table 4. Except for the Pakistani group, no chlorpyrifos contamination was observed among our samples. However, the Pakistani samples’ contamination rate was within the acceptable range of ISIRI (0.1 mg kg^−1^). Our Pakistani group presented the concentration of 0.075 ± 0.05 mg kg^−1^ presented by Almutairi et al. [33]. Hamid et al. [47] observed a higher chlorpyrifos concentration of 3.3 mg kg^−1^ and 1.45 mg kg^−1^ in two Pakistani brown rice samples.

#### 2.4.2. Trichlorfon

Another investigated insecticide, trichlorfon (metrifonate), was not detectable in Pakistani, Indian, and Thai samples; however, in the Iranian group, it did not exceed the ISIRI permissible limit (0.1 mg kg^−1^). No data were found regarding Trichlorfon contamination of rice to be compared with the present findings.

#### 2.4.3. Diazinon

This insecticide was not present in the Iranian and Thai groups, while in the Pakistani and Indian it was in the ISIRI acceptable range (0.1 mg kg^−1^). The diazinon content of the Pakistani group was significantly higher than the Indian. However, no diazinon contamination was observed in Iranian samples. Two studies from the north of Iran reported non-acceptable contamination of 0.4 ± 0.43 and 31.91 ± 31.74 mg kg^−1^ among domestic rice samples [48,49].

#### 2.4.4. Fenitrothion

This insecticide was observed only in the Iranian group. However, it was below the ISIRI permissible limit (0.05 mg kg^−1^). Regarding this insecticide, no documented data concerning its presence among rice samples were available.

#### 2.4.5. Chlorothalonil

This acaricide and fungicide were only detectable in the Pakistani group, which was below the ISIRI permissible limit (0.05 mg kg^−1^). Additionally, López-Dávila et al. [50] observed no chlorothalonil contamination in their rice sample in Cuba. Generally, the most appropriate status of pesticide contamination belonged to Thai samples with no detectable concentration of pesticides.

### 2.5. Non-Carcinogenic Health Risk Assessment

**Figure 1:**
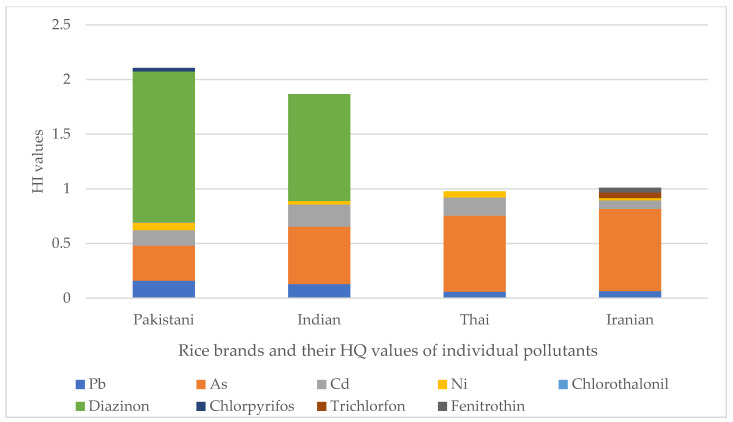
HI values of rice brands.

**Figure 2:**
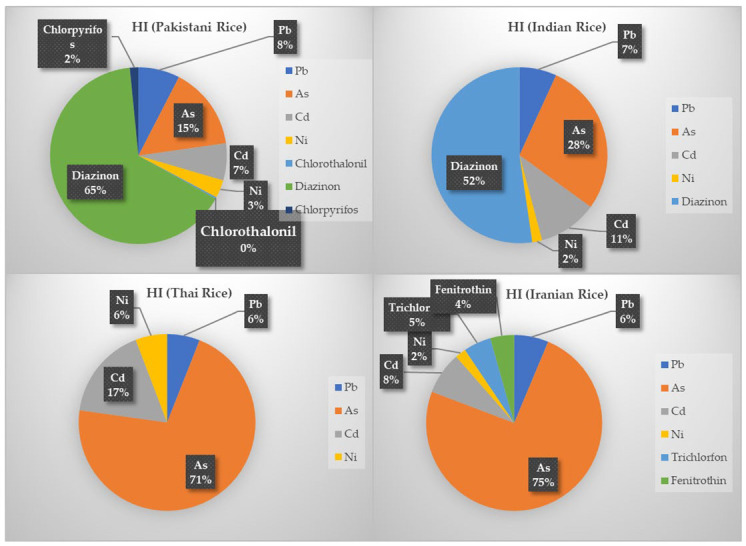
Shares of individual pollutants (%) in HI of different rice brands.

All details about EDI, HQ, and HI values are presented in Table 5 and Figure 1 and Figure 2. Among metal(loid)s of the Pakistani group, Ni and As had the highest and the lowest EDI with 1.4 × 10^−3^ and 9.6 × 10^−5^ mg kg^−1^ day^−1^, respectively. Additionally, among the pesticides, chlorpyrifos and diazinon had the highest and the lowest EDI with 1.2 × 10^−4^ and 6.6 × 10^−5^ mg kg^−1^ day^−1^. The EDI of DON and AFB1 was also estimated to be 9.4 × 10^−5^ and 7.0 × 10^−6^ mg kg^−1^ day^−1^ in Pakistani rice, respectively. Additionally, there is no daily intake of trichlorfon, fenitrothin, ZEN, and OTA due to the consumption of Pakistani rice. Regarding the HQ values of Pakistani rice pollutants, it can be claimed that the highest hazardous consumption of Pakistani rice is diazinon with 1.38, followed by As with 3.2 × 10^−1^. The lowest HQ value also belonged to chlorothalonil with 6.2 × 10^−3^. Therefore, according to the present findings, the diazinon content of Pakistani rice, with an HQ value ≥ 1, possesses a potential non-carcinogenic health risk for the Iranian adult consumer. Additionally, the HQ values from other pollutants, despite being below 1, were not so low that their relatively high levels led to an increase in HI value to about 2.1. This index for Pakistani rice is more than twice the allowable limit (HI < 1). As mentioned earlier, the biggest and smallest shares of contribution in the HI value belonged to diazinon, As, and chlorothalonil with 65.3%, 15.1%, and 0.3%.

Between the metal(loid)s content of the Indian group, Ni and As with 6.8 × 10^−4^ and 1.6 × 10^−4^ mg kg^−1^ day^−1^ had the highest and the lowest EDI, respectively. Additionally, among its pesticides, diazinon, with 4.7 × 10^−5^ mg kg^−1^ day^−1^, was the only pollutant with measurable EDI. Regarding the mycotoxins, only the ZEN and DON with EDI of 1.9 × 10^−5^ and 1.2 × 10^−5^ mg kg^−1^ day^−1^ were measurable, respectively. In this group, no daily intake of chlorothalonil, chlorpyrifos, trichlorfon, fenitrothin, and AFB1 due to consumption of Indian rice was documented. The HQ values of diazinon with 9.7 × 10^−1^, followed by As with 5.2 × 10^−1^, and Ni with 3.4 × 10^−2^ were the highest and the lowest. Although these HQ values were individually below 1, as in the Pakistani group, their high amounts combined led to an HI of 1.86, nearly twice the permissible level. The largest contributors to this HI value were the diazinon and As, with 52.3 and 28.2%, respectively, and the smallest, with 1.8%, was Ni. Similar to the Pakistani group, the consumption of Indian rice by the Iranian adult population can involve non-carcinogenic health risks.

Among the Thai group pollutants, the highest and the lowest EDI values belonged to Ni and Cd with 1.1 × 10^−3^ and 1.6 × 10^−4^ mg kg^−1^ day^−1^. Because of the absence of pesticides in Thai rice, they had no daily intake. However, the order of the EDI values of DON, ZEN, and AFB1 were 8.9 × 10^−5^, 2.2 × 10^−5^, and 5.1 × 10^−6^ mg kg^−1^ day^−1^, respectively. Additionally, there was no intake from OTA. Regarding HQ and HI, it was concluded that from Indian rice, only the HQ values related to metal(loid)s contributed to the HI of 0.98. The highest contributor metal(loid)s in HI were the As and Cd with 7.0 × 10^−1^ and 1.7 × 10^−1^. According to the present findings, the Thai rice investigated in the current study was the safest rice brand regarding non-carcinogenic health risk based on its HI value below 1 compared with the Pakistani, Indian, and Iranian rice. However, despite the HI below 1, Thai rice arsenic, with an HQ of about 0.70 (share of 71.2% in HI), could be the most serious pollutant of this group and necessitates adequate consideration.

The Iranian group’s highest and lowest metal(loid)s EDI values were Ni and Cd with 4.0 × 10^−4^ and 7.9 × 10^−5^ mg kg^−1^ day^−1^. The EDIs of trichlorfon, fenitrothin, ZEN, and DON were 1.02 × 10^−4^, 5.7 × 10^−5^, 2.5 × 10^−5^, and 1.8 × 10^−5^ mg kg^−1^ day^−1^, respectively. Notably, no daily intake from chlorothalonil, diazinon, and chlorpyrifos among pesticides and AFB1 and OTA from mycotoxins was confirmed. In relation to HQ and HI values resulting from the consumption of Iranian rice, it can be claimed that the highest and the lowest HQ values belonged to As and Ni with 7.5 × 10^−1^ and 2.0 × 10^−2^. The role of As in the formation of HI was more effective compared to Indian rice (share of 74.4%), and the lowest impact share of 2% belonged to Ni. All these HQ values were below 1, and, therefore, these pollutants individually contained no specific non-carcinogenic adverse effects. However, the HI value concluded from the consumption of Iranian rice was slightly more than 1 (1.01). It was very close to the HI value of Thai rice. As a result, it can be argued that the HI status of Thai and Iranian rice is much better than that of Pakistani and Indian rice, and their lifetime consumption will probably possess far fewer non-carcinogenic health risks and adverse effects to the Iranian adult population.

Regarding non-carcinogenic health risk assessment, there were some similar recent reports conducted in Iran and a neighboring country, Saudi Arabia, that suggested their population may have the same food habits as the Iranian population. The main challenge, which is simultaneously an advantage of the present study, is the need for similar studies in Iran that comprehensively cover all rice contaminants. They had only investigated the PTEs or, in fewer cases, studied pesticides. However, the mentioned study by Almutairi et al. [33] from Saudi Arabia can be a suitable study for comparison. For example, Javdan et al. [51] reported that the HQ values of Cd and As derived from the consumption of Indian rice in Hormozgan, Iran, were 1.7 × 10^−2^ and 4.9 × 10^−1^, respectively. Another local study from Iranshahr, Iran, also reported an HI value of 6.67 among frequently consumed rice brands, and its biggest contributor was the As, with an HQ value of 5.23 (share of 78.41% in HI). These values are much higher than our findings. However, it is noticeable that they also investigated Al, Cu, and Mo (molybdenum). They also still needed to assess the status of pesticides and Ni in their samples [34]. Sharafi et al. [5], similar to our Thai and Iranian samples, also presented the As as the biggest contributor in HI value formation among Iranian, Pakistani, and Indian samples frequently consumed in Tehran, Iran. They reported that the HQ value for As in their Iranian, Pakistani, and Indian samples was 5.65 × 10^−1^, 5.1 × 10^−1^, and 8.47 × 10^−1^, respectively. They also suggested HI values of about 1.4 for the Indian and less than 1 for the Iranian and Pakistani samples. However, they included no pesticides in their HQ and HI calculation, which can be one of our sources of difference. The most similar study to our study was the study conducted by Almutairi et al. [33] from Saudi Arabia, which in addition to heavy metals, included pesticides; however, their pesticides were different from the present study. They suggested a HI value of 3.3 × 10^−1^ in which metal(loid)s had the highest contribution to HI value with 97.4% (inorganic As 80.4%: Cd 10.7%: Pb 3.6%: Hg 3.3%). In comparison, pesticides (such as buprofezin 0.6%, malathion 0.45%, and carbendazim 0.3%) had only a 2.6% share of contribution among Indian rice consumed by the adult population of Saudi Arabia. Regarding pesticides, there was a study focused on diazinon in Iranian domestic rice cultivated in Rasht, Iran. They selected diazinon because it was considered to be frequently consumed in rice cultivation. Ghanbari et al. [48] reported a range of 5.4 to 17.2 (mean of 10.2) for the diazinon-associated health risk index (HRI) in different zones of Rasht, Iran. Their estimation was much higher than our findings.

### 2.6. Carcinogenic Health Risk Assessment

All EDI and ILCR values were calculated and reported in Table 5. Due to a lack of adequate information in terms of CSF, ILCR was calculated for only metal(loid)s and chlorothalonil.

In the Pakistani group, Ni, Pb, and As with ILCR of 9.7 × 10^−4^, 1.4 × 10^−4^, and 1.1 × 10^−4^; Cd with 4.2 × 10^−5^; and chlorothalonil with 5.5 × 10^−7^ were considered as unacceptable within the maximum acceptable range and with negligible carcinogenic risk, respectively.

In terms of the Indian group, Ni, As, and Pb with an ILCR of 4.8 × 10^−4^, 1.8 × 10^−4^, and 1.1 × 10^−4^ and Cd with 5.9 × 10^−5^ were unacceptable and within the maximum acceptable range of carcinogenic risk, respectively.

Among the Thai group, Ni and As with ILCRs of 7.9 × 10^−4^ and 2.4 × 10^−4^ and also Pb and Cd with ILCRs of 5.0 × 10^−5^ and 4.9 × 10^−5^ possessed an unacceptable carcinogenic risk and a carcinogenic risk within the maximum acceptable range, respectively.

Regarding the Iranian group, Ni and As with ILCRs of 2.8 × 10^−4^ and 2.6 × 10^−4^ and Pb and Cd with ILCRs of 5.5 × 10^−5^ and 2.3 × 10^−5^ were considered unacceptable and within the maximum acceptable range of carcinogenic health risk. Therefore, it generally seems that Ni and As, with a lesser extent of Pb, is considered potential risk in terms of carcinogenic risks derived from the consumption of four types of rice: Pakistani, Indian, Thai, and Iranian, and it necessitates that continuous monitoring and efforts for reducing them to be on the agenda. Djahed et al. [34] reported a mean ILCR of 2.4 × 10^−3^ for As, which was higher than our results. Salehipour et al. [52] presented an ILCR of more than 1 × 10^−4^ related to As from Isfahan, Iran. In addition, Sharafi et al. [5] suggested ILCR values of 2.5 × 10^−4^, 2.3 × 10^−4,^ and 3.8 × 10^−4^ for As between Iranian, Pakistani, and Indian rice types. However, Pirsaheb et al. [53] reported ILCR values of 1.42 × 10^−5^, 1.7 × 10^−5^, and 2 × 10^−6^ for As, Cd, and Pb among rice samples. Almutairi et al. [33] also presented the As-associated ILCR of 1.2 × 10^−4^. They suggested more acceptable ILCR values of 1.5 × 10^−6^–1.3 × 10^−5^ for Cd, Pb, and malathion.

## 3. Conclusions

The present study’s findings showed that although the rice samples met most of the required standards, the non-carcinogenic risk of diazinon in Pakistani and Indian rice must be considered. It is noticeable that, As in Thai and Iranian rice was the most hazardous pollutant. However, Thai and Iranian rice with an HI near 1 possessed much lower health risks in comparison with Pakistani and Indian rice. Additionally, the carcinogenic risk of Ni and As due to the consumption of domestic rice and all three other imported brands is another critical finding of this study. This associates stricter standards and special attention to more appropriate planting, harvesting, and storage conditions of rice to achieve lower levels of metals, pesticides, and mycotoxins in rice. However, fortunately, the present study’s findings show a suitable and acceptable situation regarding the presence of different mycotoxins in domestic and imported rice. However, there is still a need to maintain and improve the current situation.

## 4. Materials and Methods

### 4.1. Chemicals, Reagents, and Apparatuses

Sodium hydroxide, dichloromethane, sulfuric acid, potassium chloride, hydrochloric acid, anhydrous sodium sulfate, sodium chloride, sodium hydrogen phosphate acetone, chloroform, *n*-hexane, nitric acid, phosphate-buffered saline, acetic acid, Rose-Bengal chloramphenicol agar were purchased from Merck Company (Darmstadt, Germany); methanol and acetonitrile were supplied from Sigma-Aldrich (St. Louis, MO, USA). Deionized water was prepared using a WP750 water purifier (PG Iinstruments, Leicestershire LE17 5BH, England). The standards of PTEs (1000 μg mL^−1^), pesticides, and mycotoxins standard solutions were provided by Merck Company (Darmstadt, Germany). Electronic balance (KEB5003 model, Shanghai, China); centrifuge (CE06-M.50ee, Model: D.G.X 16D. Isfahan, Iran); oven (Fan Azma Gostar, Tehran Iran); furnace (Fan Azma Gostar, Tehran, Iran); autoclave (REIHAN TEB, RT-1 model, TYPE 4, Tehran, Iran); laminar air flow class2 (LF550, Shiraz, Iran); Whatman filter paper, grade 1, atomic absorption instrument equipped with a graphite furnace, flame, and hydride vapor generator (pg. instrument, AA500AFG model, England) and HPLC System (Agilent 1290 infinity lc); RF-10AXL fluorescence detector; LC column (Genesis C18, 250 ×4.6 mm, five μm, Radnor, PA, USA); the flow rate was 2 mL min^−1^ equaling to one drop per second; a gas chromatography (Agilent Technologies 5793 N, Santa Clara, CA, USA) instrument equipped with an auto-injector (7683B series); electron capture detector (320 °C); and column: DB-5 MS (30 m × 0.25 mm i.d.; 0.25 µm) were used.

### 4.2. Sample Collection and Study Design

In the present study, 60 samples of rice, including 15 samples of each Pakistani, Indian, Thai, and Iranian, were collected from March 2018 to March 2019 and sent to the reference laboratory. The samples selected among the food commodities were submitted to the other laboratories by the food importers, local store owners, or the farmers in Bushehr, Iran. Their ash, moisture, and mold content, mycotoxins (AFB1, OTA, ZEN, and DON), the concentration of PTEs (Pb, As, Cd, and Ni), and pesticides residues (chlorothalonil, diazinon, chlorpyrifos, trichlorfon, and fenitrothion) were examined and compared with the acceptable ranges of Institute of Standards and Industrial Research of Iran (ISIRI). According to the concluded data, a health risk assessment (carcinogenic and non-carcinogenic) was performed [11,54].

### 4.3. Measurement of Moisture, Ash, and Mold Content

Measurement of moisture and ash was performed according to AOAC [55] and Ho et al. [56]. Additionally, to determine the mold count, 1 mL of the sample was pour-plated in Rose-Bengal chloramphenicol agar (Merck, Darmstadt, Germany) and then incubated at 22 ± 1 °C for 5–6 days. Finally, mold colonies were counted [57].

### 4.4. Preparation, Sample Digestion, and Determination of PTEs

Preparation and digestion of samples and measurement of their PTEs were performed according to AOAC, 2000, using 6 M hydrochloric acid (1 + 1) and 0.1 M nitric acid solution. The same procedure was used for the preparation of blank solutions. An atomic absorption instrument with a graphite furnace, flame, and hydride vapor generator (pg. instrument, AA500AFG model, England) was used for analysis. After that, based on national standards, preparation of the standard ion solution in different concentrations was carried out, and the calibration curve was drawn for each PTE. The accuracy of the analytical procedures was verified by analysis of appropriate certificated reference materials (CRMs) using the same digestion and analytical methods. The details about validation parameters, including LOD and LOQ, are presented in Table 6. Calibration standard curves were prepared using standard solutions of a stock standard solution of cadmium (r^2^ = 0.97), arsenic (r^2^ = 0.93), lead (r^2^ = 0.98), and nickel (r^2^ = 0.95) [54,58,59].

### 4.5. Mycotoxins Determination

#### 4.5.1. Aflatoxin B1

Iranian National Standard no. 6872 was employed to measure Aflatoxin B1 using HPLC [60,61]. Separation, detection, and determination were conducted using a reversed-phase column, Kobra cell method, and fluorescence detector, comparing the area under the standard curve with an unknown sample and measuring the dilution coefficient. The rice samples were ground, and 50 g of them was mixed with 5 g of NaCl and 200 mL of 80% methanol. The resulting extract was passed through Whatman filter paper grade 1, and 20 mL of the filtered extract was mixed with 130 mL of water and passed through fiberglass filter paper with a mesh of 1.6 μm; 75 mL of the extract was given through the immunoaffinity column for Aflatoxins at the speed of 2 mL/min equivalent to one drop per second. After collecting the residual extract, 1500 μL of methanol was passed through the column and collected in a vial. Its content was diluted with 1500 μL of water and vortexed properly. Two hundred working standard solution was injected into the HPLC system. The calibration curve was plotted by calculating the standard changes in toxin mass (ng) on the *X*-axis and the changes in the area under the curve on the *Y*-axis. Then, 100 μL of the final extract was injected into the HPLC system. The contamination rate was calculated using the calibration curve, comparing the height of standard curves or the area under the curve with unknown samples, and using the dilution coefficient to detect aflatoxin B1, a fluorescence detector with an excitation wavelength of 365 nm and emission of 435 nm was employed.

#### 4.5.2. Ochratoxin

Iranian National Standard no. 9238 was used to measure ochratoxin [62]. Separation, detection, and determination via HPLC were the same with aflatoxin B1, with the difference being that a fluorescence detector with an excitation wavelength of 333 nm and emission of 477 nm was used here. An amount of 25 g of the grounded rice was mixed with 1 g of NaCl and 100 mL of solvent extraction of ochratoxin for three minutes. Then, the resulting extract was passed through Whatman filter paper grade 1. An amount of 50 mL of PBS solution and 10 mL of the filtered extract were mixed, and the diluted extract passed through fiberglass filter paper with mesh of 1.6 μm. An amount of 50 mL of the extract was passed through the specific column for ochratoxins at 2 mL/min. An amount of 1.5 mL of methanol-acetic acid with a 98 to 2 was passed through the column to wash the toxin connected with the antibody inside the column. Then, it was collected in a vial and diluted with 1.5 mL of water. An amount of 100 μL of working standard solution was injected into the HPLC system for toxin measurement. Then, the calibration curve was drawn. After that, 100 μL of the final extract was injected into the HPLC, and the contamination rate was calculated, as mentioned previously.

#### 4.5.3. Zearalenone

Iranian National Standard no. 9239 measured the zearalenone [63]. Twenty-five rounded rice were mixed with 1 g of NaCl and 100 mL of solvent extraction of zearalenone for three minutes. The resulting extract was passed through the Whatman filter paper grade 1. An amount of 10 mL of the filtered extract was diluted with 65 mL of water and passed through fiberglass filter paper with mesh of 1.6 μm. An amount of 65 mL of the filtered extract was passed through the immunoaffinity column with antibodies specific for zearalenone at the speed of one drop per second. An amount of 2 mL of methanol was passed through the column in order to wash the toxin connected with the antibody and collected in a clean vial. The vial content was diluted with 2 mL of water and vortexed. The ultraviolet detector at a wavelength of 275 nm or fluorescence detector with an excitation wavelength of 275 nm and emission of 450 nm were used here. An amount of 200 μL of zearalenone working standard solution was injected into the HPLC and the calibration curve was drawn to measure the toxin. Then, 150 μL of the final extract was injected into the HPLC. The contamination rate was calculated, as previously described.

#### 4.5.4. Deoxynivalenol

Iranian National Standard no.10215 was used to measure the deoxynivalenol [64]. An amount of 25 g of the grounded rice was mixed with 1 g of NaCl and 100 mL of DON solvent extraction for three minutes. The resulting extract was passed through Whatman filter paper grade 1, and 5 mL of the filtered extract was passed through DONSPE columns and collected in a vial. An amount of 2.5 mL of 84% acetonitrile solution was passed through the column and assembled in the vial for washing the column. The vial was left in a bain-marie at 40–50 °C and allowed to dry in the air pressure provided by a vacuum pump. After that, 1 mL solvent of deoxynivalenol mobile phase was added to the vial and vortexed for 1 min. The content of the vial was mixed by vortex for another 1 min. An amount of 50 μL of deoxynivalenol working standard solution was injected into the HPLC, and the calibration curve was drawn. Afterward, 200 μL of the final extract was injected into the HPLC. Deoxynivalenol was detected by many ultraviolet detectors at a wavelength of 218 nm. The obtained peaks were compared to the standard peak, and the contamination rate was calculated as expressed earlier.

### 4.6. Pesticides Determination

Extraction and cleanup procedure were performed according to the method described by ISIRI using gas chromatography [54]. The column temperature started from 80 °C and was kept at this temperature for 2 min. It was then set at 30 °C per min to reach 180 °C. It was held at this temperature for 2 min and then adjusted at 5 °C per min until it went to 290 °C and was kept at this temperature for 8 min. Helium gas with a constant flow of 1 mL min^−1^ was used. For injection at 250 °C, the splitless mode was employed.

### 4.7. Health Risk Assessment

To evaluate non-carcinogenic health risks derived from the consumption of these four types of rice, estimated daily intake (EDI), hazard quotient (HQ), and hazard index (HI) were calculated according to USEPA [65] and the following Equations (1)–(3).

(1) $$\mathrm{EDI}=\frac{C\times \mathrm{EF}\times \mathrm{ED}\times \mathrm{IR}}{\mathrm{AT}\times \mathrm{BW}}$$

(2) $$\mathrm{HQ}=\frac{\mathrm{EDI}}{\mathrm{ADI}}$$

(3) $$\mathrm{HI}=\sum_{i=1}^{n}\mathrm{HQi}$$

EDI (mg kg^−1^ day^−1^) was calculated based on the following indices. C (individual pollutant concentration based on mg/g dry weight of rice), EF (exposure frequency based on 365 days year^−1^); ED (exposure duration based on 54 mean years of adult Iranian population), IR (ingestion rate based on 165 g/d from Iranian per capita of rice consumption [65]), AT (average exposure time based on 54 years × 365 days for non-carcinogenic EDI and 70 years × 365 days for carcinogenic EDI), and finally BW (body weight of adult Iranian people based on 77.45 kg) [66,67,68]. After calculating the EDI for each pollutant, they were employed to be compared with acceptable daily intake values (ADI) for each pollutant. All the available RfD (reference dose) values were presented in Table 7 [65,69,70,71]. After the HQ_s_ calculation, if they are equal to or below 1, no non-carcinogenic dietary health risks would exist. At the same time, if they are higher than 1, they could be a potential source of non-carcinogenic health risks [5,33]. To evaluate the cumulative non-carcinogenic information of mentioned pollutants, the HI value for each type of rice was calculated with the summation of their entire specific HQs. The same interpretation applies to HI as it does to HQ.

To assess the carcinogenic health risks, EDI for each pollutant was also calculated, as it was performed for non-carcinogenic risk with only a difference in the amount of AT mentioned previously. After that, the following Equation (4) incremental lifetime cancer risk (ILCR) was calculated.

(4) ILCR=EDI (non−carcinogenic)×CSF

In the above equation, CSF is the cancer slope factor, and it is based on the mg kg^−1^ day^−1^. All the available CSFs are reported in Table 7. Based on the USEPA definition, ILCR values between 10^−8^ and 10^−6^, 10^−6^ and 10^−4^, and more than 10^−4^ are considered to be negligible, maximum acceptable limit, and unacceptable carcinogenic health risks, respectively [33,65].

### 4.8. Statistical Analysis

Each parameter of rice types was analyzed separately using the analysis of variance (ANOVA) test. Mean values ± standard error (SE) were reported for each case. The study was performed using the SPSS software (SPSS 16 for Windows, SPSS Inc., Chicago, IL, USA). The *p* values less than 0.05 were considered statistically significant.

## Figures and Tables

**Table 1:** Ash, moisture, and mold contamination.

Parameter	Pakistan	India	Thailand	Iran
Moisture (%)	8.801 ± 0.288 ^b^	10.691 ± 0.286 ^a^	9.893 ± 0.458 ^ab^	9.273 ± 0.411 ^b^
Ash (%)	0.788 ± 0.322 ^a^	0.58 ± 0.025 ^b^	0.662 ± 0.049 ^b^	0.563 ± 0.036 ^b^
Mold (log CFU/g)	2.94 ± 0.34 ^d^	4.11 ± 0.58 ^a^	3.6 ± 0.48 ^b^	3.21 ± 0.41 ^c^

Small letters indicate significant differences in a row and between all treatments, respectively (*p* < 0.05).

**Table 2:** Heavy metals contamination.

Heavy Metal	Limit of National Standard (mg kg^−1^)	Pakistan	India	Thailand	Iran
As	0.15	0.045 ± 0.019 ^b^	0.074 ± 0.014 ^ab^	0.098 ± 0.014 ^a^	0.106 ± 0.020 ^a^
Pb	0.15	0.30 ± 0.08 ^a^	0.24 ± 0.06 ^ab^	0.11 ± 0.03 ^b^	0.12 ± 0.04 ^b^
Cd	0.06	0.067 ± 0.007 ^b^	0.095 ± 0.011 ^a^	0.078 ± 0.006 ^ab^	0.037 ± 0.007 ^c^
Ni	-	0.650 ± 0.054 ^a^	0.320 ± 0.035 ^c^	0.53 ± 0.03 ^b^	0.19 ± 0.039 ^d^

Different letters in each column characterize significant differences at a level of *p* < 0.05.

**Table 3:** Mycotoxin contamination.

Mycotoxins	Pakistan	India	Thailand	Iran	Limit of National Standard (µg kg^−1^)
Aflatoxin B1 (AFB1) (µg kg^−1^)	3.3 ± 0.04 ^a^	<LOD	2.4 ± 0.01 ^b^	<LOD	5
Zearalenone (ZEN) (µg kg^−1^)	<LOD	8.8 ± 0. 44 ^b^	10.3 ± 0.25 ^ab^	11.8 ± 0.32 ^a^	200
Ochratoxin A (OTA) (µg kg^−1^)	<LOD	<LOD	<LOD	<LOD	5
Deoxynivalenol (DON) (µg kg^−1^)	44 ± 0.88 ^b^	55 ± 1.7 ^a^	42 ± 2.54 ^b^	38 ± 2.33 ^c^	1000

Different letters in each column characterize significant differences at a level of *p* < 0.05.

**Table 4:** Pesticides residue.

Pesticides	Limit of National Standard (mg kg^−1^)	Pakistan	India	Thailand	Iran
Chlorpyrifos	0.1	0.056 ± 0.08 ^a^	nd	nd	nd
Trichlorfon	0.1	nd	nd	nd	0.048 ± 0.05 ^a^
Diazinon	0.1	0.031 ± 0.03 ^a^	0.022 ± 0.01 ^b^	nd	nd
Fenitrothion	0.05	nd	nd	nd	0.027 ± 0.006 ^a^
Chlorothalonil	0.05	0.044 ± 0.005 ^a^	nd	nd	nd

Different letters for each heavy metal show significant differences at *p* < 0.05. nd—not detected.

**Table 5:** Health risk assessment (carcinogenic and non-carcinogenic).

Rice Brands and Pollutants		EDI_non-carcinogenic_ (mg kg^−1^ day^−1^)	HQ	EDI_carcinogenic_ (mg kg^−1^ day^−1^)	ILCR
Pakistani	Pb	6.4 × 10^−4^	1.6 × 10^−1^	4.9 × 10^−4^	1.4 × 10^−4left^
	As	9.6 × 10^−5^	3.2 × 10^−1^	7.4 × 10^−5^	1.1 × 10^−4^
	Cd	1.4 × 10^−4^	1.4 × 10^−1^	1.1 × 10^−4^	4.2 × 10^−5^
	Ni	1.4 × 10^−3^	6.9 × 10^−2^	1.1 × 10^−3^	9.7 × 10^−4^
	Chlorothalonil	9.4 × 10^−5^	6.2 × 10^−3^	7.2 × 10^−5^	5.5 × 10^−7^
	Diazinon	6.6 × 10^−5^	1.4		
	Chlorpyrifos	1.2 × 10^−4^	3.3 × 10^−2^		
	Trichlorfon				
	Fenitrothion				
	AFB1	7.03 × 10^−6^			
	ZEN				
	OTA				
	DON	9.4 × 10^−5^			
HI_Pakistani_			2.1		
Indian	Pb	5.1 × 10^−4^	1.2 × 10^−1^	3.9 × 10^−4^	1.1 × 10^−4^
	As	1.6 × 10^−4^	5.3 × 10^−1^	1.2 × 10^−4^	1.8 × 10^−4^
	Cd	0.2 × 10^−3^	2.0 × 10^−1^	1.6 × 10^−4^	5.9 × 10^−5^
	Ni	6.8 × 10^−4^	3.0 × 10^−2^	5.3 × 10^−4^	4.8 × 10^−5^
	Chlorothalonil				
	Diazinon	4.7 × 10^−5^	9.7 × 10^−1^		
	Chlorpyrifos				
	Trichlorfon				
	Fenitrothion				
	AFB1				
	ZEN	1.9 × 10^−5^			
	OTA				
	DON	1.2 × 10^−5^			
HI_Indian_			1.86		
Thai	Pb	2.4 × 10^−4^	6.0 × 10^−2^	1.8 × 10^−4^	5.1 × 10^−5^
	As	2.1 × 10^−4^	7.0 × 10^−1^	1.6 × 10^−4^	2.4 × 10^−4^
	Cd	1.6 × 10^−4^	1.7 × 10^−1^	1.3 × 10^−4^	4.9 × 10^−5^
	Ni	1.1 × 10^−3^	6.0 × 10^−2^	8.7 × 10^−4^	7.9 × 10^−4^
	Chlorothalonil				
	Diazinon				
	Chlorpyrifos				
	Trichlorfon				
	Fenitrothion				
	AFB1	5.1 × 10^−6^			
	ZEN	2.2 × 10^−5^			
	OTA				
	DON	8.9 × 10^−5^			
HI_Thai_			0.98		
Iranian	Pb	2.6 × 10^−4^	6.0 × 10^−2^	1.9 × 10^−4^	5.5 × 10^−5^
	As	2.3 × 10^−4^	7.5 × 10^−1^	1.7 × 10^−4^	2.6 × 10^−4^
	Cd	7.9 × 10^−5^	8.0 × 10^−2^	6.1 × 10^−5^	2.3 × 10^−5^
	Ni	4.1 × 10^−4^	2.0 × 10^−2^	3.1 × 10^−4^	2.8 × 10^−4^
	Chlorothalonil				
	Diazinon				
	Chlorpyrifos				
	Trichlorfon	1.02 × 10^−4^	5.0 × 10^−2^		
	Fenitrothion	5.7 × 10^−5^	4.0 × 10^−2^		
	AFB1				
	ZEN	2.5 × 10^−5^			
	OTA				
	DON	1.8 × 10^−5^			
HI_Iranian_			1.01		

**Table 6:** Pollutants quality assurance parameters.

Component	Wavelength (nm)	Sensitivity (mg/L)	LOQ	LOD	Recovery (%)	R^2^
As	193.7	0.40	0.032 (mg kg^−1^)	0.012 (mg kg^−1^)	91	0.93
Pb	283.3	0.18	0.055	0.022	87	0.98
Cd	228.8	0.012	0.01	0.008	93	0.97
Ni	232	0.08	0.06	0.015	89	0.95
Chlorpyrifos			0.2 (mg kg^−1^)	0.01 (mg kg^−1^)	91	
Trichlorfon			0.2	0.01	84	
Diazinon			0.3	0.02	88	
Fenitrothion			0.2	0.01	87	
Chlorothalonil			0.1	0.02	89	
AFB1			0.85 (µg kg^−1^)	0.2 (µg kg^−1^)	88.6	0.993
ZEN			13	8.5	90.3	0.994
OTA			0.65	0.3	81	0.992
DON			34.8	22.44	84	0.991

LOQ: limit of quantification, LOD: limit of detection.

**Table 7:** RfD and CSF values.

Contaminants	Reference Dose (RfD) (mg kg^−1^ day^−1^)	Cancer Slope Factor (CSF) (mg kg^−1^ day^−1^)
Pb	4 × 10^−3^	2.8 × 10^−1^
As	3 × 10^−4^	1.5
Cd	1 × 10^−3^	3.8 × 10^−1^
Ni	2 × 10^−2^	9.1 × 10^−1^
Chlorothalonil	1.5 × 10^−2^	7.66 × 10^−3^
Diazinon	4.8 × 10^−5^	
Chlorpyrifos	3.6 × 10^−3^	
Trichlorfon	2 × 10^−3^	
Fenitrothion	1.3 × 10^−3^	

## Data Availability

All data generated or analyzed during this study are included in this published article.
